# The Influence of Hydroxyl Group on Nerve Excitability Blockade by Limonene and Its Hydroxylated Metabolites, Perillyl Alcohol and Carveol

**DOI:** 10.3390/molecules31152732

**Published:** 2026-08-06

**Authors:** Lívia Carolina Amâncio, Edvanildo de Sousa-Silva, André Nogueira Cardeal-dos-Santos, Isabella Soares Marques Rabelo, Gustavo Paes de Andrade Saraiva, Ana Carolina Cardoso-Teixeira, Maria Diana Moreira-Gomes, José Ednésio da Cruz Freire, Andrelina Noronha Coelho-de-Souza, Francisco Walber Ferreira-da-Silva, Kerly Shamyra da Silva-Alves, José Henrique Leal-Cardoso

**Affiliations:** 1 Laboratory of Electrophysiology, Superior Institute of Biomedical Sciences, State University of Ceará, Av. Silas Munguba 1700, Campus of Itaperi, Fortaleza 60714-903, CE, Brazil; carol.amancio143@gmail.com (L.C.A.); edvanildo7@gmail.com (E.d.S.-S.); isabellasoaresmed@gmail.com (I.S.M.R.); gustavopaes0306@gmail.com (G.P.d.A.S.); anacarolina.acct@gmail.com (A.C.C.-T.); dianammgr@hotmail.com (M.D.M.-G.); walber_ferreira@uvanet.br (F.W.F.-d.-S.); kerly.shamyra@uece.br (K.S.d.S.-A.); 2 Laboratory of Experimental Physiology, Superior Institute of Biomedical Sciences, State University of Ceará, Fortaleza 60714-903, CE, Brazil; andrecardealdossantos@gmail.com (A.N.C.-d.-S.); andrelina.noronha@uece.br (A.N.C.-d.-S.); 3 Biochemistry and Gene Expression Laboratory, Superior Institute of Biomedical Sciences, State University of Ceará, Fortaleza 60714-903, CE, Brazil; jednesio@gmail.com; 4 Technological and Exact Science Center, State University Vale do Acaraú, Sobral 62040-730, CE, Brazil

**Keywords:** limonene, perillyl alcohol, carveol, sciatic nerve, excitability, compound action potential, nerve conduction

## Abstract

A previous investigation on limonene (LM), perillyl alcohol (POH), and carveol (CV), focused on the structure–activity relationship and hydroxyl group, documented that the presence of the hydroxyl group influences the pharmacodynamic potency of these agents, inhibiting smooth muscle contraction with the order of potency: POH > CV > LM. That investigation also suggested a mechanism of action, which importantly included activity on the voltage-dependent calcium channel. Here, we investigated whether this structure–activity relationship also applies to nerve excitability (an activity greatly dependent on sodium channels) using compound action potential (CAP) recordings from mouse sciatic nerves and in silico simulations. POH, CV, and LM inhibited both the positive amplitudes and conduction velocities of the two CAP components in a concentration-dependent manner, with IC_50_ values of 0.8, 1.0, and 4.3 mM (1st component) and 0.6, 0.6, and 3.0 mM (2nd component) for amplitude, and 2.4, 2.4, and 7.1 mM (1st component) and 1.0, 2.6, and 4.3 mM (2nd component) for conduction velocity. The order of pharmacodynamic potency, thus, was POH = CV > LM. In silico simulation demonstrated that POH and CV penetrate the Nav 1.6 and accommodate in the channel at the interface between the selectivity filter and the central cavity, a position very favorable to block the channel pore. In contrast, LM exhibited a markedly different docking profile, suggesting that LM binding is less likely to directly obstruct sodium permeation. In conclusion, the three substances investigated inhibited nerve excitability, but those with a hydroxyl group demonstrated greater pharmacodynamic potency.

## 1. Introduction

The repertoire of diseases that need better and cheaper treatment remains large. This has stimulated the search for new medicines, and for that purpose, the scientific community has been giving increasing attention to terpenes and terpenoids. This is because several of them could be easily found as natural constituents of essential oils [1,2]. This is also because of their pharmacological activities and therapeutic potentialities [1,3]. From the pharmacological point of view, they have been described as antispasmodic; antinociceptive and analgesic [4,5,6]; antihypertensive [7,8], anti-inflammatory [9,10,11,12], anesthetic [13], vasorelaxant [14,15], gastroprotective [16,17,18], and antineoplastic [11,19,20], including perillyl alcohol (POH) for treatment of the very aggressive cancer glioblastoma [21,22,23,24,25,26].

**Figure 1:**
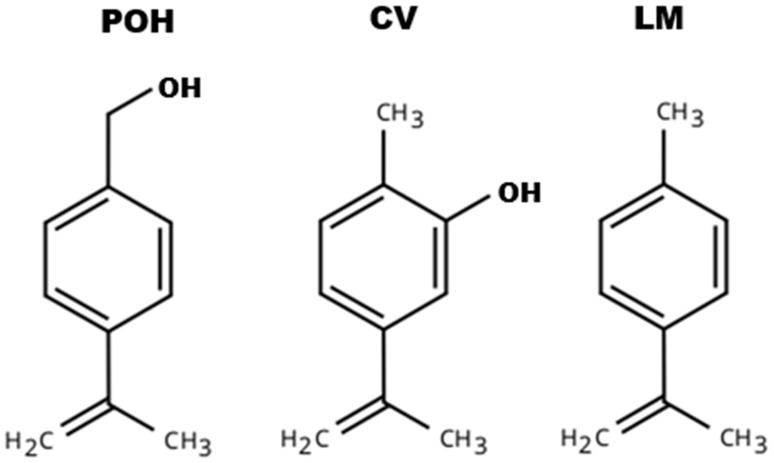
Planar chemical structure of perillyl alcohol (POH), carveol (CV), and limonene (LM). The hydroxyl group is highlighted in bold.

One trend of the pharmacology of terpenes and terpenoids that has raised increased interest is the molecular structure–activity relationship [1,27]. Terpenes and terpenoids are volatile substances of small molecular weight, usually very lipophilic. One radical that could have significant implications on the lipophilicity/hydrophilicity of the molecule is the hydroxyl [28,29,30]. Indeed, some studies have demonstrated the importance of the hydroxyl group of terpenoids in modulating pharmacological activities [5,30,31,32]. The presence and the position of polar functional groups, such as the -OH, have been demonstrated to be critical factors that can influence the pharmacological potency and efficacy of these molecules [5,33]. Investigation of our laboratory on the influence of the hydroxyl group on the inhibitory effect of POH, carveol (CV), and limonene (LM) (Figure 1) on smooth muscle contraction demonstrated that the presence and the position of the hydroxyl group may alter the pharmacological potency [5]. The previous investigation [5] used POH and CV, with the hydroxyl group attached to a primary and secondary carbon atom, respectively, and LM, without hydroxyl group. The pharmacological potencies differed significantly and were POH > CV > LM [5]. The inhibitory effect of the three substances was interpreted to be mainly through the effect on the voltage-dependent Ca^2+^ channel.

One important aspect of the contribution of the hydroxyl group to the pharmacological effects of monoterpenes is its influence on nerve excitability. Peripheral nerves are composed of distinct fiber populations that differ in their electrophysiological properties and susceptibility to pharmacological modulation. Recording the compound action potential (CAP) provides a simple and reliable electrophysiological approach for assessing changes in the excitability of these different types of nerve fibers. Therefore, to investigate whether perillyl alcohol (POH), carveol (CV), and limonene (LM) modulate nerve excitability, and to determine the extent to which the hydroxyl group influences the pharmacodynamic parameters underlying this effect, we evaluated their actions on the compound action potential of the mouse sciatic nerve.

In addition to electrophysiological investigations, computational approaches have become valuable tools for elucidating the molecular determinants underlying the interaction of monoterpenes with voltage-gated ion channels [34,35,36,37,38,39,40,41]. Techniques such as molecular docking, molecular dynamics simulations, and binding free energy calculations provide atomistic insights into ligand binding modes, interaction stability, and the contribution of specific functional groups to receptor affinity. In this context, in silico analyses offer an opportunity to clarify how the presence and stereochemical position of the hydroxyl group in POH and CV, compared with the non-hydroxylated LM, influence their interactions with voltage-gated Na^+^ channels, thereby providing a mechanistic explanation for the experimentally observed differences in pharmacological potency [42,43,44,45,46,47,48].

Thus, this study was designed to bridge experimental pharmacology and computational biophysics by investigating the interactions of POH, CV, and LM with voltage-gated Na^+^ channels at the molecular level. We hypothesized that differences in the presence and stereochemical orientation of the hydroxyl group would alter ligand binding, interaction stability, and energetic favorability, ultimately accounting for the distinct inhibitory potencies observed experimentally. Integrating electrophysiological data with molecular simulations is expected to provide a comprehensive mechanistic understanding of the structure–activity relationships governing the effects of these monoterpenes.

## 2. Results

### 2.1. Effect of Perillyl Alcohol on CAP Amplitude and Velocity of Conduction

**Figure 2:**
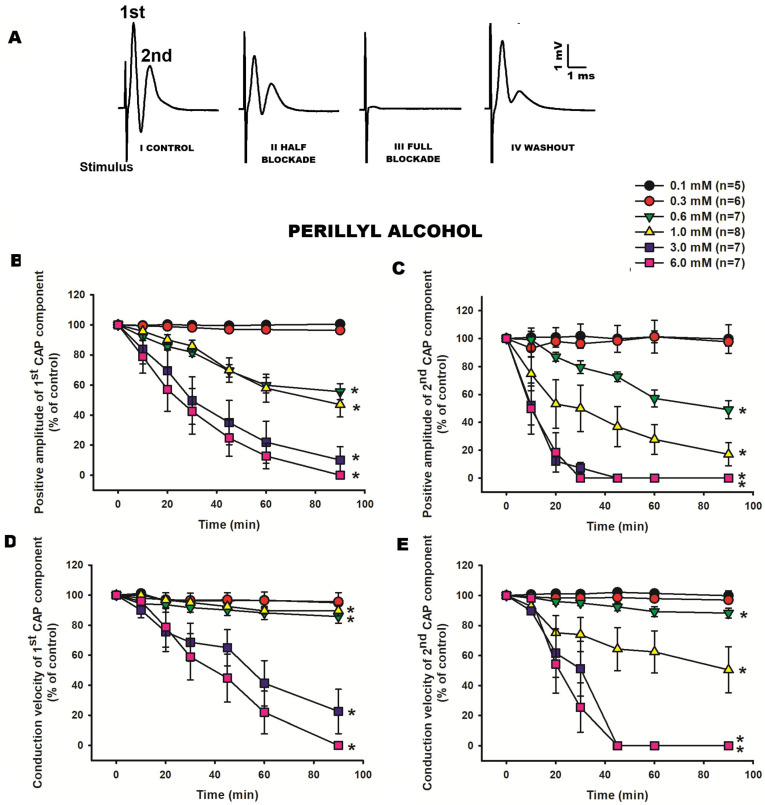
Time course of inhibitory effect of perillyl alcohol (POH) on components of compound action potential (CAP) of mouse sciatic nerve. (**A**) Representative tracings showing the progressive action of POH (3 mM) on the CAP, at the beginning (I), at approx. 50% inhibition (II), at approx. 100% inhibition (III), and at 90 min after washout (IV). Panels (**B**,**C**) show the time course of the inhibitory effects of POH on the positive amplitudes of the first and second CAP components, respectively. Panels (**D**,**E**) show the effects of POH on the conduction velocities of the first and second CAP components, respectively. Data are presented as mean ± S.E.M., and the “n” for each concentration is indicated in the legend. * *p* < 0.05 compared with the control (ANOVA followed by Dunn’s test).

POH (0.1–6.0 mM) had a depressive effect on the amplitude and velocity of conduction of CAP components (Figure 2). For smaller concentrations, for the first and second CAP components, this inhibitory effect on excitability developed slowly. Concerning amplitude (mean control value for nerves used with POH were 3.59 ± 0.93 and 1.43 ± 0.75 mV, respectively, for the first and second CAP components), 0.6 mM was the threshold concentration for blockade of the positive amplitude of both CAP components (Figure 2B,C). Concerning the velocity of conduction (mean control value 57.20 ± 5.93 and 19.24 ± 6.64 m/s, respectively, for the first and second CAP components), 0.3 and 0.6 mM were threshold concentrations for the velocity of conduction of first and second components, respectively (Figure 2D,E). The POH effect was reversible upon washout. 

### 2.2. Effect of Carveol on CAP Amplitude and Velocity of Conduction

**Figure 3:**
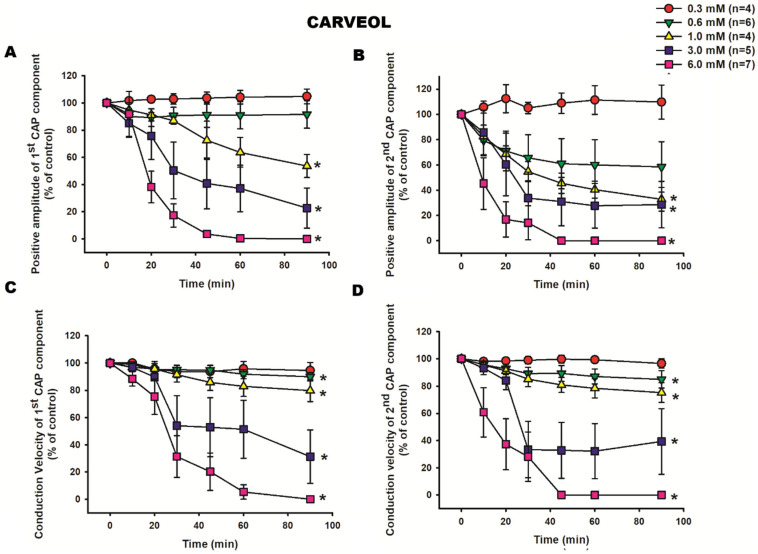
Time course of the inhibitory effects of carveol (CV) on the components of the compound action potential (CAP) of the mouse sciatic nerve. Panels (**A**,**B**) show the time course of the inhibitory effects of CV on the positive amplitudes of the first and second CAP components, respectively. Panels (**C**,**D**) show the effects of CV on the conduction velocities of the first and second CAP components, respectively. Data are presented as mean ± S.E.M., and the “n” for each concentration is indicated in the legend. * *p* < 0.05 compared with the control (ANOVA followed by Dunn’s test). No representative CAP trace for CV is shown because its profile was similar to that observed for POH and therefore did not provide additional relevant information.

CV promoted effects on the CAP of the sciatic nerve of the rat that were similar to those induced by POH. CV (0.3–6.0 mM) depressed nerve excitability and inhibited the amplitude and velocity of conduction of CAP components (Figure 3). Concerning amplitude, 1.0 mM was the threshold concentration for the inhibition of both CAP components (Figure 3A,B). Regarding the velocity of conduction, 0.6 mM was the threshold concentration for both components’ inhibition (Figure 3C,D). For nerves treated with CV, mean control values for amplitude were 2.83 ± 0.36 and 1.46 ± 0.31 mV for first and second components, respectively; those for conduction velocity were 56.49 ± 8.23 and 24.77 ± 8.20 m/s. 

### 2.3. Effect of Limonene on CAP Amplitude and Velocity of Conduction

**Figure 4:**
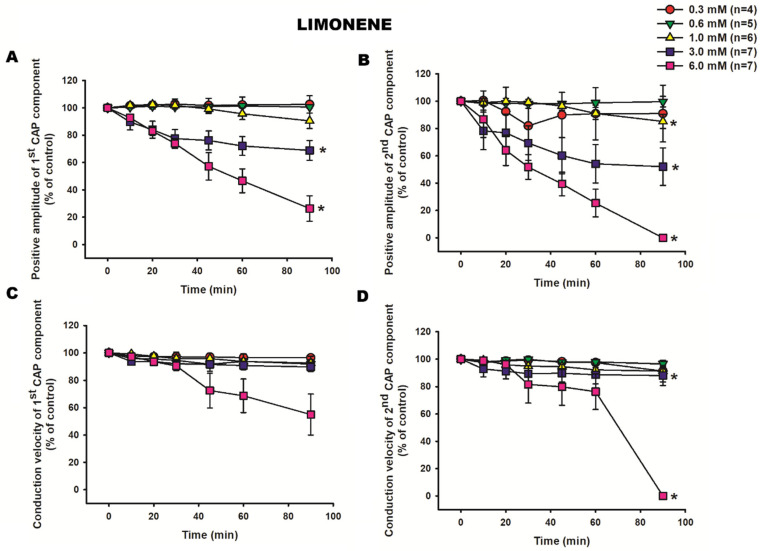
Time course of the inhibitory effects of limonene (LM) on the components of the compound action potential (CAP) of the mouse sciatic nerve. Panels (**A**,**B**) show the time course of the inhibitory effects of LM on the positive amplitudes of the first and second CAP components, respectively. Panels (**C**,**D**) show the effects of LM on the conduction velocities of the first and second CAP components, respectively. Data are presented as mean ± S.E.M., and the “n” for each concentration is indicated in the legend. * *p* < 0.05 compared with the control (ANOVA followed by Dunn’s test). No representative CAP trace for LM is shown because its profile was similar to that observed for POH and therefore did not provide additional relevant information.

**Figure 5:**
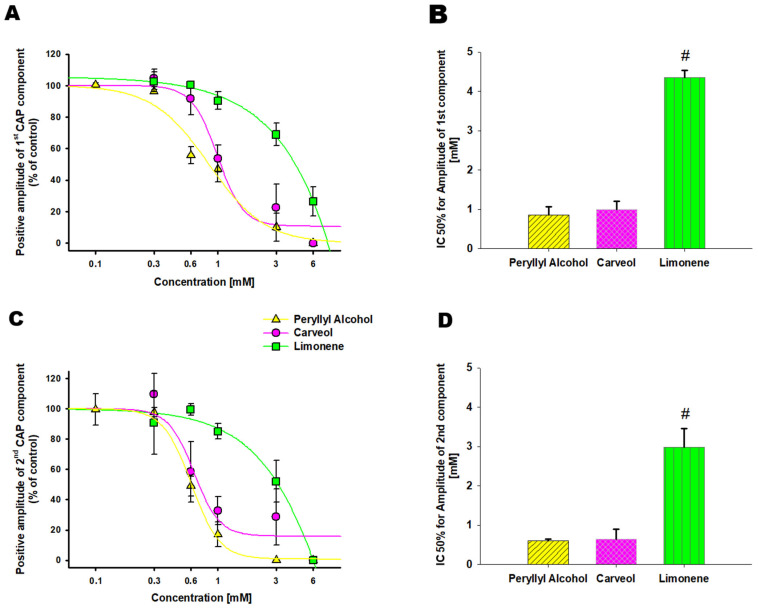
Concentration-dependent inhibition of the amplitude of CAP components at the end of 90 min of preparation exposure to perillyl alcohol (POH), carveol (CV), and limonene. (**A**,**C**) Concentration–response curves showing the normalized amplitude of the first and second components, respectively, plotted against the concentration (mM) of POH (yellow triangles), CV (purple circles), and LM (green squares). Solid lines represent non-linear regression fits (sigmoidal dose–response). (**B**,**D**) Bar graphs displaying the mean half-maximal inhibitory concentration (IC50) values for the first (**B**) and second (**D**) components. Data are presented as mean ± S.E.M. # indicates a statistically significant difference compared to the POH and CV groups (*p* < 0.05; one-way ANOVA and Bonferroni *t*-test).

**Figure 6:**
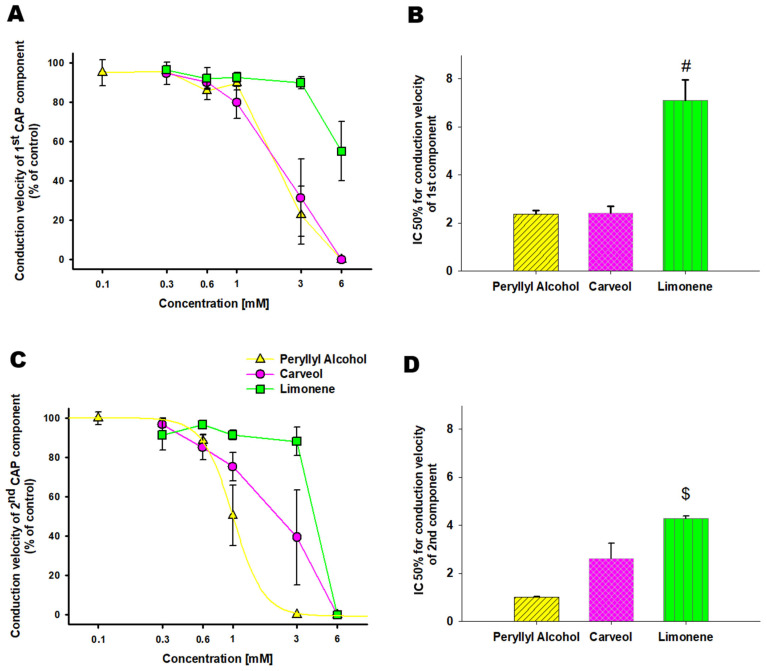
Effects of LM, CV, and POH on the conduction velocity of the first and second components. (**A**,**C**) Concentration–response curves showing the relative conduction velocity of the first (**A**) and second (**C**) components plotted against the concentration (mM) of LM (green squares), CV (purple circles), and POH (yellow triangles). Solid lines represent non-linear regression fits. (**B**,**D**) Bar graphs displaying the mean IC50 values for the first (**B**) and second (**D**) components. Data are presented as mean ± S.E.M. # indicates a statistically significant difference compared to the POH and CV groups, while $ indicates a statistically significant difference compared to the POH group (*p* < 0.05; one-way ANOVA and Bonferroni *t*-test).

LM (0.3–6.0 mM) also had a concentration-dependent inhibitory activity on the amplitude and velocity of conduction of CAP. For the first component, the threshold concentration for significant inhibition was 3.0 and 6.0 mM, for amplitude and velocity of conduction, respectively; for the second component, the respective values were 1.0 and 3.0 mM. For nerves treated with CV, mean control values for amplitude were 3.79 ± 0.39 and 1.97 ± 0.52 mV for the first and second components, respectively; those for conduction velocity were 41.55 ± 4.34 and 29.39 ± 8.34 m/s. The LM IC50 for amplitude or velocity of conduction of CAP are shown in Figure 4, Figure 5 and Figure 6. It was concerning the pharmacodynamic parameters that LM showed a great difference from POH and CV. Concerning pharmacodynamic efficacy, it reached maximal values (100% blockade) for POH and CV, first and second CAP components; for LM, it was maximal for inhibition of amplitude and velocity of conduction of the second CAP component but not for inhibition of the first component. Something similar occurred to pharmacodynamic potency: POH and CV had IC_50_s that, for each parameter and component measured, showed a magnitude that was smaller than the respective LM value. This configures, for LM, a smaller pharmacodynamic potency than that of POH and CV. It also configures, for the first CAP component, pharmacodynamic efficacy, which was smaller for LM. The POH, CV and LM effects were reversible upon washout.

### 2.4. Binding Profiles of Monoterpenes in the Nav1.6 Pore

**Figure 7:**
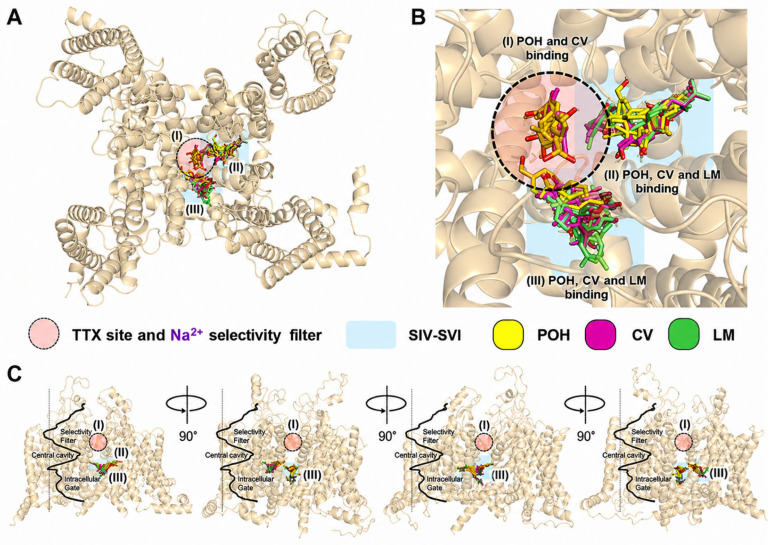
Identification of three recurrent binding regions for POH, CV, and LM within the human Nav1.6 sodium channel. (**A**) Overall structure of the Nav1.6 channel showing the distribution of docking poses for POH (yellow), CV (magenta), and LM (green). Three preferential binding regions were identified (I–III). The tetrodotoxin (TTX) binding site and Na^+^ selectivity filter are highlighted in pink, while the SIV–SVI fenestration is shown in light blue. (**B**) Enlarged view of the three binding regions. Region I is predominantly occupied by POH and CV, whereas Regions II and III accommodate poses from all three ligands (POH, CV, and LM). (**C**) Orthogonal views (90° rotation) illustrating the spatial relationship between the three binding regions and the channel architecture, including the extracellular vestibule, selectivity filter, central cavity, and intracellular gate. The results indicate that POH and CV preferentially occupy the ion conduction pathway near the selectivity filter and central cavity, whereas LM predominantly localizes to peripheral hydrophobic regions outside the pore, indicating distinct binding modes. The yellow, green and magenta colored ligands are carbon atoms and red colored ligands are oxygen atoms.

In silico simulation analysis of molecular interactions of POH, CV and LM with the voltage-gated sodium channel Nav1.6 were done (Figure 7). A total of 20 independent runs were conducted for each ligand, yielding a set of interaction poses. Structural analysis revealed that POH and CV were successfully accommodated within the ion conduction pathway, specifically targeting the interface between the selectivity filter and the central cavity. Conversely, LM showed a profile interaction of channels very different from POH and CV. LM failed to establish stable interactions or convergent poses within the pore module, predominantly docking to non-specific hydrophobic pockets outside the permeation pathway, since LM docking positions were all of them outside DEKA locus components of channel pore.

**Figure 8:**
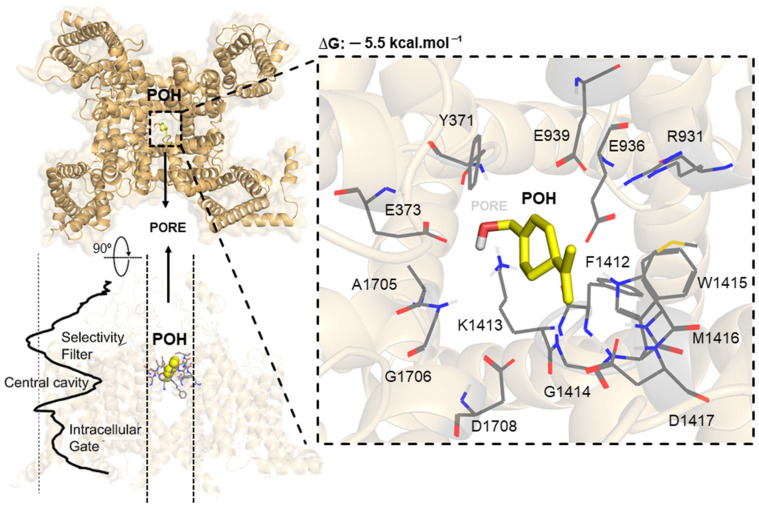
Molecular docking and binding energetics of POH within the Nav1.6 pore. Structural overview of the Nav1.6–POH complex. The left panel localizes the interaction site at the interface between the selectivity filter and the central cavity. The expanded view (right) details the binding microenvironment, where the POH ligand (yellow sticks) establishes intermolecular contacts with key residues, including E373, E936, E939, and K1413. The calculated free energy of binding ∆G, −5.5 kcal·mol^−1^, indicating favorable stabilization via hydrophobic and electrostatic interactions in the inner pore region. The grey/yellow colored residues/ligands are carbon atoms, red colored residues are oxygen, the blue residues are nitrogen atoms.

POH exhibited a favorable binding affinity with a free energy (∆G) = −5.5 kcal·mol^−1^ (Figure 8). The ligand anchored itself deep within the pore, flanked by domains DI, DII, and DIV. The binding mode is stabilized by a network of interactions involving residues critical for ion selectivity, including the DEKA locus components. Specifically, the hydroxyl group of POH is oriented towards a polar cluster, establishing interactions with Y371, E373, E936, and E939. Concurrently, the hydrophobic terpene ring engages in Van der Waals contacts with F1412, W1415, M1416, and the selectivity filter residue K1413.

**Figure 9:**
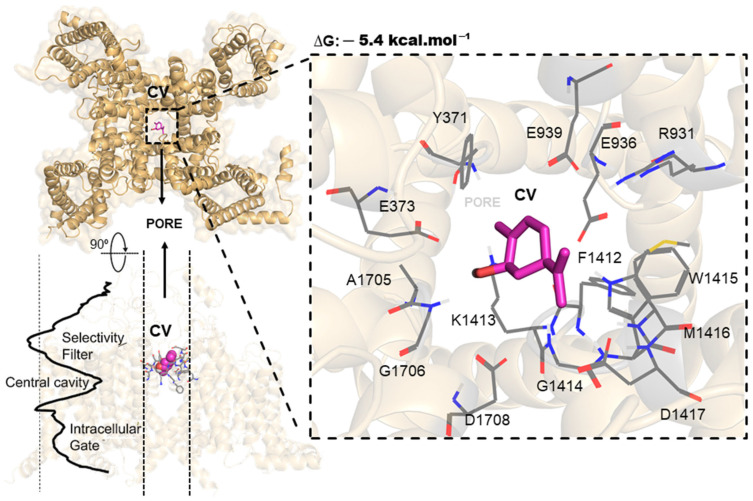
Molecular docking and binding energetics of CV within the Nav1.6 pore. Structural overview of the Nav1.6–CV complex. The left panel localizes the interaction site at the interface between the selectivity filter and the central cavity. The expanded view (right) details the binding microenvironment, where the POH ligand (magenta sticks) establishes intermolecular contacts with key residues, including E373, E936, E939, and K1413. The calculated free energy of binding ∆G: −5.4 kcal·mol^−1^, indicating favorable stabilization via hydrophobic and electrostatic interactions in the inner pore region. The grey/magenta colored residues/ligands are carbon atoms, red colored residues are oxygen, the blue residues are nitrogen atoms.

CV exhibited a binding profile spatially like POH, docking into the central cavity (CV site) with a binding energy of ∆G = −5.4 kcal·mol^−1^. The ligand interacts extensively with the inner pore architecture, surrounded by residues from the four homologous domains. Key stabilizing residues include Y371, E939, and R931, which form a coordinate network around the ligand. Notably, CV is positioned near the intracellular gate residues and the DEKA locus components, such as K1413 and A1705 (Figure 9).

## 3. Discussion

The major discovery of the present investigation is that POH, CV, and LM, both at 0.3–6.0 mM range, exhibit inhibitory activity on nerve excitability, a finding that is novel for CV. However, POH, and CV do it with different pharmacodynamics as compared to LM. POH and CV, molecules bearing a hydroxyl group, showed greater pharmacodynamic potency and, as related to the first component, greater pharmacodynamic efficacy than LM, a molecule without a hydroxyl group. This finding, related to a probable effect on the Na+ channel, confirms the tendency observed by other investigations that the presence of a hydroxyl group in the molecule increases the pharmacodynamic parameters of potency and efficacy. This work is pioneering in portraying the differences between these monoterpenes in relation to their chemical structure and nerve excitability, as observed through the lenses of the pharmacodynamic characteristics of their effects on CAP parameters of the sciatic nerve.

This study highlights the important role of the hydroxyl group (-OH) on the pharmacological activity of these small molecules. The incorporation of the -OH group, as analyzed in POH and CV, confers greater polarity and amphipathicity to the molecule, as compared to LM (it is a purely lipophilic precursor). This structural modification not only potentiates the bioactive effects known in the literature, such as neuroprotective and antioxidant properties via activation of the Nrf2/HO-1 pathway, but also increases hydrosolubility, which agrees with previous research [5,6,49,50,51,52]. These physicochemical characteristics favor greater bioavailability and efficacy, as observed, for example, for POH [53], establishing hydroxylation as a fundamental chemical determinant to enhance the action of these compounds in biological systems.

The electrophysiological results of this study provide powerful evidence of the impact of the hydroxyl group on the interaction with ion channels and neuronal excitability. The ability of hydroxylated monoterpenes to induce a faster and more intense blockade of the CAP suggests a mechanism of action similar to that of local anesthetics, involving the modulation of voltage-gated sodium channels and/or potassium channels, as observed by other authors [15,54,55,56]. While LM, due to its lipophilicity, can act non-specifically, altering membrane fluidity, the amphipathic nature of POH and CV allows for a more specific and stable binding to polar amino acids within or near the ion pore [5]. This selective interaction is important to confer greater blocking efficacy, demonstrated by CAP suppression at lower concentrations and in a shorter time compared to LM. Another study observed the effect of carvone (10 mM) on the CAP of mouse sciatic nerves for 30 min; the results showed a partial blockade of the CAP amplitude and velocity. The authors emphasize the importance of the hydroxyl group in the structures of CV and carvacrol for the blockade of the CAP [32].

Comparative analysis of the blocking effects revealed the following order of potency and onset of action: POH = CV > LM; this underscores the importance of the hydroxyl group for the inhibitory activity of these monoterpenes. Although POH and CV are both alcoholic monoterpenes, they differ in the position of the hydroxyl group, which is attached to a primary carbon in POH and to a secondary carbon in CV. This structural difference was previously shown to influence their effects on smooth muscle contraction [5], but it did not appear to affect their ability to suppress nerve excitability. This apparent discrepancy may reflect differences in their primary molecular targets. In smooth muscle, the effects are likely mediated mainly through voltage-operated calcium channels (VOCCs), or possibly other components of the contractile machinery [5], whereas in peripheral nerves, the most probable target is the voltage-gated Na^+^ channel [57].

The rapid onset of CAP inhibition observed with POH and CV is consistent with a state-dependent mechanism of channel block, in which the compounds preferentially interact with the open or inactivated states of voltage-gated Na^+^ channels. This mechanism is well established for local anesthetics and several other ion channel modulators [56]. It is therefore plausible that the molecular conformation of POH and CV enhances the steric accessibility of the hydroxyl group to channel binding sites [14,47,55], promoting a more stable interaction with the inactivated state of the channel and, consequently, a faster suppression of action potential propagation.

The potency observed for POH and CV is consistent with previous reports demonstrating that oxygenated monoterpenes are generally more effective inhibitors of nerve excitability and voltage-gated sodium channels than non-oxygenated analogues. Carvacrol has been reported to inhibit native sodium currents in dorsal root ganglion neurons with an IC_50_ of approximately 0.37 mM [47], while thymol inhibits voltage-gated sodium channels with IC_50_ values ranging from 104 to 149 μM, depending on the channel subtype [58]. Menthol exhibits lower potency, with IC_50_ values between 376 and 571 μM. Although direct quantitative comparisons should be interpreted cautiously because of differences in experimental preparations and sodium channel isoforms, these findings collectively support the notion that oxygenated functional groups are important determinants of sodium channel inhibition, in agreement with the structure–activity relationship identified in the present study.

An interesting finding was the greater pharmacological potency observed on the second CAP component, corresponding to Aδ fibers, compared with the first component, which reflects conduction in Aα/β fibers. Although the mechanisms underlying this differential sensitivity were not investigated in the present study, one possible explanation is that the larger, heavily myelinated Aα/β fibers possess a higher safety factor for impulse conduction. Consequently, a greater degree of sodium channel blockade may be required to impair action potential propagation in these fibers than in the smaller Aδ fibers, rendering the latter more susceptible to conduction failure under pharmacological inhibition [59].

### 3.1. Convergent Molecular Mechanisms of Pore Blockade by POH and CV

Comparative analysis of molecular docking reveals that POH and CV share a similar binding mode within the Nav1.6 central cavity, exploiting a pore architecture rich in polar and charged residues, whilst LM does not. Although both terpenes exhibit comparable binding energies (∆G = −5.5 kcal·mol^−1^), the stabilization of each ligand is mediated by a specific network of contacts with amino acids crucial for ion conductance in the case of ionic channels (Figure 7). The similar docking scores obtained for POH and CV indicate comparable energetic stabilization once bound to the channel. However, SAR analysis indicates that the critical determinant of biological activity is not the docking energetic score itself, but the hydroxyl group, which promotes productive polar interactions and proper orientation within the pore. In contrast, the absence of this functional group in LM limits its ability to achieve a more pore specific blocking binding mode, consistent with its lower experimental potency.

### 3.2. Interaction with the Acidic Cluster

A fundamental structural determinant for the anchoring of both compounds is the interaction with the cluster of acidic residues located in Domains I and II [60]. Our simulations indicate that both POH and CV are positioned to form hydrogen bonds and electrostatic interactions with Glu373 and Glu393 [57]. These residues, which coordinate the guanidinium and hydroxyl groups of the toxin in the TTX complex, act here as polar anchoring points for the hydroxyl (-OH) group of the monoterpenes. Glu936, part of the DEKA locus responsible for sodium selectivity, also participates in stabilization, suggesting that the physical presence of these ligands directly disrupts the primary coordination site of the Na^2+^ ion [61].

### 3.3. Role of K1413 and Filter Occlusion

Additionally, residue Lys1413 (Domain III), another critical component of the selectivity filter, plays a central role in the proposed blocking mechanism [62]. Both, POH and CV, project their cyclic structures toward the side chain of Lys1413. This interaction contributes to the physical steric barrier and potentially interferes with the electrostatic repulsion necessary for rapid cation permeation [63]. The proximity to Lys1413, also observed in the TTX binding mode, reinforces the hypothesis that these terpenes occlude the permeation pathway at a vital constriction point [64].

### 3.4. Hydrophobic and Conformational Stabilization

Beyond polar interactions, both ligands are stabilized by Van der Waals contacts with conserved aromatic and hydrophobic residues, notably Phe1412 and Trp1415 [13]. Structural comparison suggests that while TTX acts as a high-affinity electrostatic “plug” in the extracellular vestibule, POH and CV function as blockers that penetrate deeper into the cavity, anchored by the acidic residues (E373, E936, E939) and the filter lysine (K1413) [61]. This mechanism partially mimics the spatial occupancy of the classic toxin but operates through distinct binding chemistry based on the amphipathicity of the oxygenated terpenes.

The findings of the present study further support the concept that structure–activity relationship analysis represents a valuable strategy for the rational development of neuroactive agents derived from monoterpenes. Hydroxylation appears to be a key determinant of pharmacological potency and tissue selectivity in excitable tissues, in agreement with previous studies [5]. In particular, the optimized polarity and molecular conformation of POH and CV enhance their ability to interact more efficiently and with faster kinetics with ion channels, resulting in superior activity compared with LM. This mechanistic insight provides a rational framework for the structural optimization of natural compounds, as previously demonstrated for molecules such as 1,8-cineole, carvacrol, and thymol [14,55], ultimately supporting the development of novel therapeutic agents with improved efficacy, bioavailability, and pharmacodynamic profiles, particularly for the modulation of neuronal excitability. Overall, the blocking mechanism exhibited by POH and CV appears to be mediated by the anchoring of the hydroxyl group to amino acid residues located within the central cavity and the selectivity filter of the Nav1.6 channel, thereby stabilizing channel blockade and reducing neuronal excitability. In contrast, LM did not show pore specific combination in blocking binding mode.

## 4. Materials and Methods

### 4.1. Animals and Tissue Preparation

Sixty-four Swiss mice (*Mus musculus*) of both sexes, weighing 18–20 g, were obtained from UniChristus vivarium and maintained with free access to food and water under controlled conditions (22–26 °C; 12 h light/dark cycle) at the Animal Facility of the State University of Ceará (UECE). All procedures were approved by the Committee for Ethical Use of Experimental Animals of UECE (protocol number 10510522.2021).

Both sciatic nerves were dissected after the euthanasia of the animal and nourished with modified Locke’s solution, containing (in mM) NaCl 140, KCl 5.6, MgCl_2_ 1.2, CaCl_2_ 2.2, Tris (hydroxymethylaminomethane) 10, and glucose 10, pH adjusted to 7.4 ± 0.01 (10–12 °C). After dissection, the nerve was positioned over electrodes in a recording chamber initially filled with Locke’s solution. A segment of nerve positioned between the stimulating and recording electrodes was maintained in contact with the solution to ensure tissue viability and drug delivery. The chamber was covered to avoid the desiccation of tissue. and all experiments were carried out at room temperature (range 22–26 °C).

### 4.2. Drugs Preparation

POH, CV, and LM were diluted in Locke’s solution containing DMSO (0.02% *v*/*v*, final vehicle (DMSO) dilution). The animals’ sciatic nerves were treated with five concentrations (0.3, 0.6, 1, 3, and 6 mM) for CV and LM, whereas for POH, an additional concentration (0.1 mM) was included, totaling six concentrations. An experimental series was run with only the vehicle, and no effect on the electrophysiological recording was detected during 90 min of nerve exposure to the vehicle (external control). Four to eight nerves were used for each concentration of each substance in the majority of substance concentrations. Finally, for each concentration of a given substance, a single nerve from a single animal was used. Thus, no concentration was tested more than once in a nerve obtained from the same animal.

All substances employed were of analytical grade, purchased from Sigma Chemical Company (St. Louis, MO, USA).

### 4.3. Compound Action Potential Recording

The CAP was recorded as previously described [65]. Briefly, the sciatic nerve was positioned in the chamber as described above, and its proximal extremity was stimulated with 40 V, 0.1 ms pulses delivered at a frequency of 0.2 Hz. The CAP was registered in the distal extremity of the nerve. The experimental protocol was divided into two sequential periods: (1) Stabilization and control period: CAP recording was typically performed for 90 min, during which the nerve was exposed only to Locke solution without additives. During this period, it was verified that no changes occurred in the peak-to-peak amplitude of the CAP during at least the last 30 min (internal control). (2) Experimental period: After the internal control period (CT), the nerve was exposed to a given concentration of one of the drugs for 90 min. After this period, washout was performed, and additional minutes of observation were allowed to verify at least partial recovery.

The CAP typically presented at least two waves (CAP components), and the parameters related to excitability and conductibility that were evaluated included the positive component amplitude and conduction velocity of each component.

### 4.4. Molecular Docking and System Preparation

The structures of the human voltage-gated sodium channel Nav1.6 were retrieved from the Protein Data Bank (PDB). The structural coordinates corresponding to the macromolecule in the apo state (PDB ID: 8GZ1) and the complex with the pore blocker 4,9-anhydro-tetrodotoxin (PDB ID: 8GZ2) were used as templates [57]. Molecular docking simulations were performed using AutoDock Tools v.1.5.6 and AutoDock Vina v.1.1.2 [66]. Prior to screening, the docking protocol was validated via site-directed re-docking of the co-crystallized ligand (4,9-ah-TTX) into the Nav1.6 pore binding site, ensuring the reproduction of the experimental binding pose [67].

### 4.5. Ligand and Receptor Parametrization

The chemical structures of the monoterpenes POH (PubChem CID: 10819), CV (PubChem CID: 7438), and LM (PubChem CID: 22311) were retrieved and energy-minimized [68]. For the receptor preparation, water molecules and heteroatoms were removed from the PDB: 8GZ2 structure. Polar hydrogen atoms were added to the Nav1.6 protein, and partial atomic charges were assigned according to the Gasteiger method. Similarly, ligands were parameterized with Gasteiger charges, and all torsional bonds were set to be rotatable, while the receptor backbone remained rigid [34].

### 4.6. Simulation Parameters

The search space was defined to encompass the selectivity filter and the central cavity of the channel, based on the known binding site of TTX. The grid box dimensions were set to 30 × 30 × 30 Å, centered at coordinates X: 133.036, Y: 136.675, and Z: 135.468. Docking calculations were executed with an exhaustiveness of 33, an energy range of 3, and a randomized seed of 2009. Fifty binding conformations were generated for each ligand. Post-docking visualization and interaction analysis were carried out using PyMOL v. 2.5.4 for 3D inspection, Discovery Studio 2024 ligand interaction diagrams [69].

### 4.7. Statistical Analysis

Results were expressed as mean ± SEM (n), where SEM is the standard error of the mean, and n is the total number of experiments. Statistical analysis and graph construction were performed using Sigmaplot (version 11.0, Palo Alto, CA, USA). The obtained results were evaluated by Kruskal–Wallis One-Way Analysis of Variance on Ranks, followed by Dunn’s post hoc test. Group mean values were considered significantly different when the *p*-value for the null hypothesis was less than or equal to 0.05. The IC_50_ values were determined by fitting concentration–response curves relating the amplitude and conduction velocity of the CAP components to the concentrations of the tested compounds. The Hill equation was used to fit the concentration–effect curves. When the Hill model did not provide an adequate fit, IC_50_ values were estimated by interpolation between the experimental data points closest to the assumed IC_50_.

## 5. Conclusions

The results of this study compare, for the first time, the concentration-dependent blockade of nerve excitability induced by perillyl alcohol, carveol, and limonene in the mouse sciatic nerve. This effect, likely mediated by the blockade of Nav 1.6 sodium channels, confers upon these monoterpenes a neurodepressant action similar to that of local anesthetics.

The structure–activity relationship analysis led to the most significant finding: the presence of a hydroxyl group drastically enhances both the potency and kinetics of the blockade. The monoterpene alcohols (perillyl alcohol and carveol) were significantly more potent than LM. Furthermore, the subtle structural difference between perillyl alcohol and carveol, differently from what happened with smooth muscle contraction [5], did not show relevant significance differences in pharmacodynamic parameters. In summary, this work underscores the importance of monoterpene hydroxylation as an important chemical determinant for developing neuroactive agents. Perillyl alcohol, which has very low toxicity and is already being developed for cancer therapeutic treatment, has its therapeutic potentiality increased by studies like the present one.

## Data Availability

The raw data supporting the conclusions of this article will be made available by the authors on request.
